# Nardilysin regulates inflammation, metaplasia, and tumors in murine stomach

**DOI:** 10.1038/srep43052

**Published:** 2017-02-23

**Authors:** Yuto Kimura, Kozo Ikuta, Takeshi Kimura, Tsutomu Chiba, Hiroko Oshima, Masanobu Oshima, Eiichiro Nishi, Hiroshi Seno

**Affiliations:** 1 Department of Gastroenterology and Hepatology, Kyoto University Graduate School of Medicine, 54 Shogoin-Kawahara-cho, Sakyo-ku, Kyoto, 606-8507, Japan; 2 Department of Cardiovascular Medicine, Kyoto University Graduate School of Medicine, 54 Shogoin-Kawahara-cho, Sakyo-ku, Kyoto, 606-8507 Japan; 3 Division of Genetics, Cancer Research Institute, Kanazawa University, Kakuma-machi, Kanazawa, 920-1192 Japan

## Abstract

Chronic inflammation contributes to a wide variety of human disorders. In the stomach, longstanding gastritis often results in structural alterations in the gastric mucosa, including metaplastic changes and gastric cancers. Therefore, it is important to elucidate factors that are involved in gastric inflammation. Nardilysin (N-arginine dibasic convertase; Nrdc) is a metalloendopeptidase of the M16 family that promotes ectodomain shedding of the precursor forms of various growth factors and cytokines by enhancing the protease activities of a disintegrin and metalloproteinase (ADAM) proteins. Here, we have demonstrated that Nrdc crucially regulates gastric inflammation caused by *Helicobacter felis* infection or forced expression of prostaglandin E_2_ in *K19-C2mE* mice. Metaplastic changes following gastric inflammation were suppressed by the deletion of *Nrdc*. Furthremore, the deletion of *Nrdc* significantly suppressed *N*-methyl-*N*-nitrosourea (MNU)-induced gastric tumorigenesis in the murine stomach. These data may lead to a global therapeutic approach against various gastric disorders by targeting Nrdc.

Chronic inflammation contributes to a wide variety of human disorders. In the stomach, most lifelong chronic gastritis is caused by *Helicobacter pylori* infection^1^, although other etiologies, such as non-steroidal anti-inflammatory drugs or autoimmunity, are also prevalent^2^. Longstanding gastritis can result in structural alterations in the gastric mucosa, including metaplastic changes and gastric cancers. Metaplastic changes in the stomach are associated with an increased risk of gastric cancer^3^. Therefore, it is important to elucidate factors that are involved in gastric inflammation for effective prevention of various gastric diseases, including gastric cancer^4^.

The local microenvironment influences the development of chronic gastritis and metaplastic changes regardless of causative factors, and inflammatory cytokines participate in constructing this environment. There are a number of factors regulating inflammatory cytokines. A disintegrin and metalloproteinase (ADAM) family proteins are involved in ectodomain shedding, and regulate the biological activities of structurally and functionally diverse inflammatory cytokines in a context-dependent manner^5^,^6^. Indeed, dysregulation of ectodomain shedding of those factors can be profoundly involved in the pathogenesis of a serial development of gastritis, metaplasia, and gastric cancer^7^.

Nardilysin (N-arginine dibasic convertase; NRDC), a zinc peptidase of the M16 family that selectively cleaves dibasic sites^8^, is diffusely localized in the cytoplasm, and is secreted to the cell surface by undetermined mechanisms^9^. We previously identified NRDC as a specific binding partner of heparin-binding epidermal growth factor-like growth factor (HB-EGF)^10^. NRDC also enhances the shedding of tumor necrosis factor-α (TNF-α) through activation of ADAM17^11^,^12^. TNF-α is produced as a membrane-anchored protein, shed from the cell surface by proteolytic cleavage, and subsequently activated. The proinflammatory genotype of TNF-α is associated with more than twice the risk of non-cardia gastric cancer^13^. In this respect, we previously demonstrated that NRDC regulates activation of TNF-α and subsequent production of inflammatory cytokines in gastric cancer cells^14^. These findings suggest that NRDC regulates chronic inflammation and tumorigenesis in the stomach; however, the *in vivo* role of NRDC in the stomach is still unclear.

In this study, we examined the role of Nrdc during the development of chronic gastritis and metaplastic changes in the stomach using *Nrdc* knockout mice. We also investigated the effect of Nrdc in chemically-induced gastric tumorigenesis.

## Results

### Nrdc did not alter the differentiation status of gastric mucosa under physiological conditions

**Figure 1:**
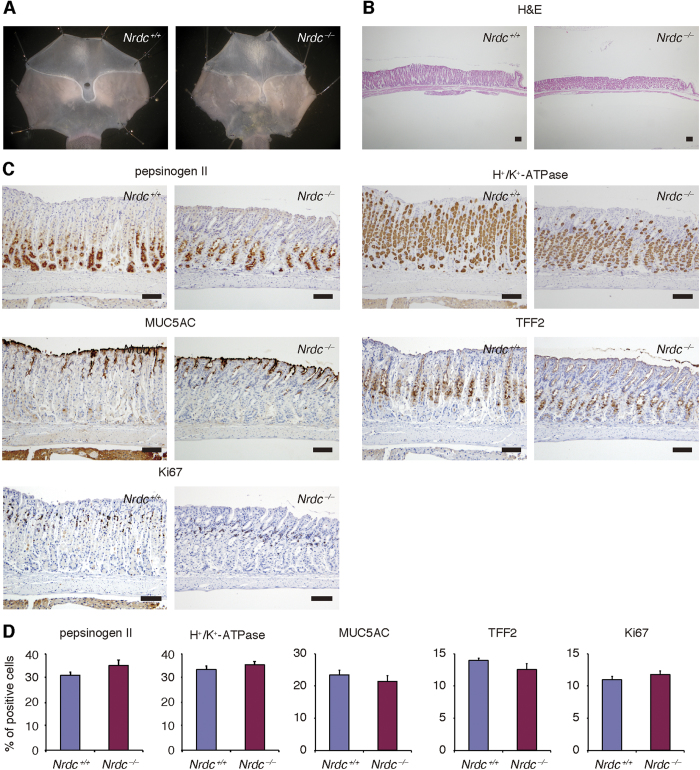
Stomachs of *Nrdc*^+/+^ and Nrdc^−/−^ mice under physiological conditions. (**A**) Representative macroscopic views of the stomachs of *Nrdc*^+*/*+^ and *Nrdc*^−/−^ mice. (**B**) H&E staining of *Nrdc*^+*/*+^ and *Nrdc*^−/−^ mouse stomachs. Bars = 100 μm. (**C**) Immunohistochemistry for pepsinogen II, H^+^/K^+^-ATPase, Muc5ac, TFF2, and Ki67 in *Nrdc*^+*/*+^ and *Nrdc*^−/−^ mice. Bars = 100 μm. (**D**) Percentages of epithelial cells immunostained with pepsinogen II, H^+^/K^+^-ATPase, Muc5ac, TFF2, and Ki67 in *Nrdc*^+*/*+^ and *Nrdc*^−/−^ mice.

We first examined the effects of *Nrdc* deletion in the stomach under physiological conditions. *Nrdc*^−/−^ mice tend to maintain lower body weights than *Nrdc*^+*/*+^ mice^15^. Therefore, the mean stomach size of *Nrdc*^−/−^ mice was slightly smaller than that of *Nrdc*^+*/*+^ mice (Fig. 1A). Other than the stomach size, there were no macroscopic differences between *Nrdc*^+*/*+^ and *Nrdc*^−/−^ mice. Although the gastric mucosa was slightly thinner in *Nrdc*^−/−^ mice (Fig. 1B), there were no significant differences in the differentiation status of gastric mucosal cells (Fig. 1C). Immunohistochemistry showed that the percentages of pepsinogen II-positive cells within gastric glands were not significantly different between *Nrdc*^+*/*+^ and *Nrdc*^−/−^ mice. This indicated that chief cell differentiation was not affected by *Nrdc* status (Fig. 1C and D). Further, immunostainings for H^+^/K^+^-ATPase, Muc5ac, and TFF2 did not show significant differences between *Nrdc*^+*/*+^ and *Nrdc*^−/−^ mice, indicating that differentiation of parietal, pit, and mucous neck cells was not regulated by Nrdc under physiological conditions, respectively (Fig. 1C and D). Furthermore, the proportion of Ki67-positive cells in the gastric glands of *Nrdc*^−/−^ mice was similar to that of *Nrdc*^+*/*+^ mice. Therefore, deletion of *Nrdc* did not alter epithelial cell proliferation under physiological conditions (Fig. 1C and D).

### Gastritis caused by *Helicobacter felis* infection was attenuated by *Nrdc* deletion

**Figure 2:**
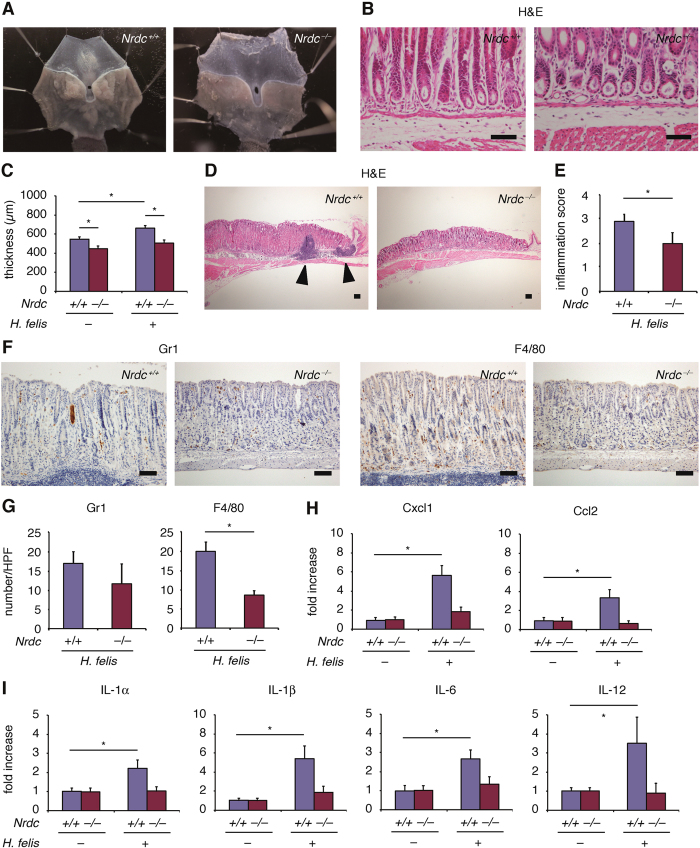
Stomachs of *Nrdc*^+/+^ and Nrdc^−/−^ mice infected with Helicobacter felis. (**A**) Representative macroscopic views of the stomachs of *Nrdc*^+*/*+^ and *Nrdc*^−/−^ mice infected with *Helicobacter felis.* (**B**) Detection of *Helicobacter felis* in the mucosae of *Nrdc*^+*/*+^ and *Nrdc*^−/−^ mice. Note that *Helicobacter felis* is detected in gastric glands of both *Nrdc*^+*/*+^ and *Nrdc*^−/−^ mice. Bars = 100 μm. (**C**) Mucosal thickness of *Nrdc*^+*/*+^ and *Nrdc*^−/−^ mice with or without *Helicobacter felis* infection. (**D**) Formation of lymphoid follicle (arrowheads) in *Nrdc*^+*/*+^ and *Nrdc*^−/−^ mouse gastric mucosae. Bars = 100 μm. (**E**) Inflammation scores of *Nrdc*^+*/*+^ and *Nrdc*^−/−^ mouse gastric mucosae. *P < 0.05. (**F**) Immunohistochemistry for Gr1 and F4/80 in *Nrdc*^+*/*+^ and *Nrdc*^−/−^ mice. Bars = 100 μm. (**G**) Number of epithelial cells immunostained for Gr1 and F4/80 in *Nrdc*^+*/*+^ and *Nrdc*^−/−^ mice. *P < 0.05. (**H**) mRNA expression of Cxcl1 and Ccl2 in the gastric mucosae of *Nrdc*^+*/*+^ and *Nrdc*^−/−^ mouse stomachs. (**I**) mRNA expression of IL-1α, IL-1β, IL-6, and IL-12 in the gastric mucosae of *Nrdc*^+*/*+^ and *Nrdc*^−/−^ mouse stomachs. *P < 0.05.

*Helicobacter felis* infection is a well-characterized mouse model that mimics chronic *Helicobacter pylori* infection in the human stomach^16^. Based on a previous report^16^, we administered suspending water containing *Helicobacter felis* to *Nrdc*^+*/*+^ and *Nrdc*^−/−^ mice for three days. Mice were sacrificed 20 weeks after the end of administration. Regardless of *Nrdc* gene status, *Helicobacter felis* was infected successfully into the gastric mucosae (Fig. 2A and B). The thickness of the mucosa of gastric corpus in *Nrdc*^+*/*+^ mice with *Helicobacter felis* infection was significantly different from mice without the infection, but was not significantly different from *Nrdc*^−/−^ mice regardless of *Helicobacter felis* infection (Fig. 2C). Of note, formation of lymphoid follicles, a characteristic of *Helicobacter felis* infection, was scarce in *Nrdc*^−/−^ mice (Fig. 2D), and the inflammation score was more severe in *Nrdc*^+*/*+^ than in *Nrdc*^−/−^ mice with *Helicobacter felis* infection (Fig. 2E). Consistent with these findings, infiltration of Gr1-positive neutrophils was not prominent in *Nrdc*^−/−^ mice, and that of F4/80-positive macrophages was significantly decreased compared to *Nrdc*^+*/*+^ mice (Fig. 2F and G). The mRNA expression of Cxcl1 and Ccl2, factors that recruit neutrophils and macrophages, respectively, was also decreased in *Nrdc*^−/−^ mice (Fig. 2H). Notably, mRNA levels of interleukin (IL)-1α, IL-1β, IL-6, and IL-12, that can contribute to chronic gastritis in humans^13^,^14^,^15^,^16^,^17^, were not significantly increased in *Nrdc*^−/−^ mice (Fig. 2I). Thus, the deletion of *Nrdc* attenuates gastric inflammation caused by *Helicobacter* infection.

### Gastritis caused by forced expression of prostaglandin (PG)E_2_ was attenuated by *Nrdc* deletion

**Figure 3:**
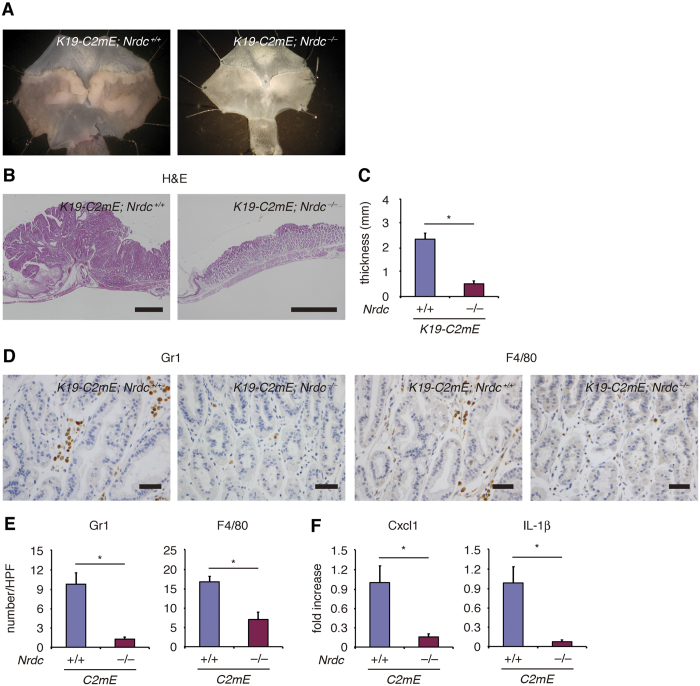
Gastritis caused by forced expression of prostaglandin E_2_. (**A**) Representative macroscopic views of the stomachs of *Nrdc*^+*/*+^ and *Nrdc*^−/−^ mice with forced expression of PGE_2_ by the insertion of *K19-C2mE* alleles. (**B**) H&E staining of *Nrdc*^+*/*+^ and *Nrdc*^−/−^ mouse stomachs with forced expression of PGE_2_. Bars = 1000 μm. (**C**) Mucosal thickness of the *Nrdc*^+*/*+^ and *Nrdc*^−/−^ mice with forced PGE_2_ expression. *P < 0.05. (**D**) Immunohistochemistry for Gr1 and F4/80 in *Nrdc*^+*/*+^ and *Nrdc*^−/−^ mice. Bars = 100 μm. (**E**) Numbers of epithelial cells immunostained for Gr1 and F4/80 in *Nrdc*^+*/*+^ and *Nrdc*^−/−^ mice. *P < 0.05. (**F**) mRNA expression of Cxcl1 and IL-1β in the gastric mucosae of *Nrdc*^+*/*+^ and *Nrdc*^−/−^ mouse stomachs. *P < 0.05.

We examined the role of Nrdc in *K19-C2mE* mice, another mouse model that expresses PGE_2_ abundantly in the gastric mucosa and mimics human gastritis^16^. Similar to *Helicobacter felis*–induced gastritis, mucosae of the gastric corpus of *Nrdc*^+*/*+^ mice were macroscopically thicker compared to *Nrdc*^−/−^ mice at 30 weeks of age (Fig. 3A). Histologically, the mucosae of gastric corpus were remarkably hyperplastic in *Nrdc*^+*/*+^ mice, consistent with a previous report^16^. However, this hyperplastic change was not prominent in *Nrdc*^−/−^ mice (Fig. 3B). Consequently, mucosae were significantly thinner in *Nrdc*^−/−^ mice (Fig. 3C). Infiltration of Gr1-positive neutrophils and F4/80-positive macrophages was significantly decreased in *Nrdc*^−/−^ mice compared to *Nrdc*^+*/*+^ mice (Fig. 3D and E). The expression of Cxcl1 and IL-1β mRNA was significantly decreased in *Nrdc*^−/−^ mice compared to *Nrdc*^+*/*+^ mice (Fig. 3F), although we could not demonstrate alterations of Ccl2, IL-1α, IL-6, and IL-12 mRNA between *Nrdc*^+*/*+^ and *Nrdc*^−/−^ mice in the presence of *K19-C2mE* alleles. Thus, like *Helicobacter felis*–induced gastritis, deletion of *Nrdc* attenuates gastric inflammation and hyperplasty caused by the forced expression of PGE_2_.

### Metaplastic changes were attenuated by *Nrdc* deletion

**Figure 4:**
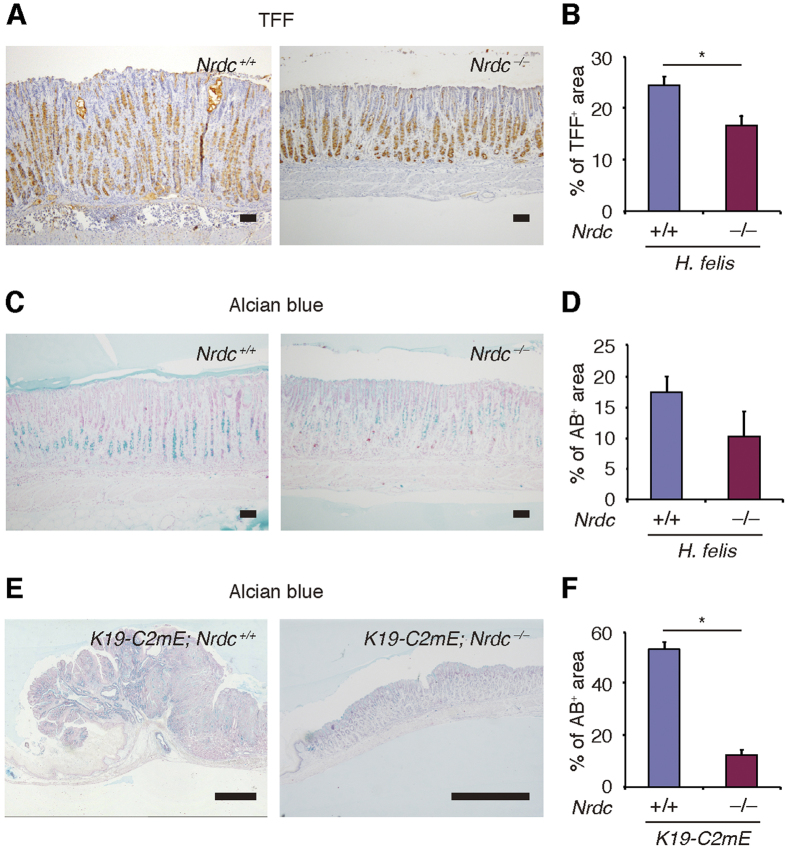
Metaplastic changes in *Nrdc*^+/+^ and Nrdc^−/−^ mouse stomachs. (**A**)Immunohistochemistry for TFF2 in *Nrdc*^+*/*+^ and *Nrdc*^−/−^ mouse stomachs with *Helicobacter felis* infection. Bars = 100 μm. (**B**) Areas stained for TFF2 in *Nrdc*^+*/*+^ and *Nrdc*^−/−^ mouse stomachs with *Helicobacter felis* infection. *P < 0.05. (**C**) Alcian blue staining of *Nrdc*^+*/*+^ and *Nrdc*^−/−^ mouse stomachs with *Helicobacter felis* infection. Bars = 100 μm. (**D**) Areas stained with Alcian blue in *Nrdc*^+*/*+^ and *Nrdc*^−/−^ mouse stomachs with *Helicobacter felis* infection. *P < 0.05. (**E**) Alcian blue staining of *Nrdc*^+*/*+^ and *Nrdc*^−/−^ mouse stomachs with PGE_2_ expression. Bars = 1000 μm. (**D**) Areas stained with Alcian blue in *Nrdc*^+*/*+^ and *Nrdc*^−/−^ mouse stomachs with forced PGE_2_ expression. *P < 0.05.

We next investigated whether Nrdc plays a role in metaplastic changes following gastritis. Staining for TFF2 and/or staining with Alcian blue are widely used to detect metaplastic changes in the gastric corpus^18^. Upon *Helicobacter felis* infection for 20 weeks, the percentage of TFF2-stained cells was significantly lower in *Nrdc*^−/−^ mice than in *Nrdc*^+*/*+^ mice (Fig. 4A and B). The percentage of Alcian blue-stained cells was also reduced in *Nrdc*^−/−^ mice compared to *Nrdc*^+*/*+^ mice at 26 weeks of *Helicobacter felis* infection (Fig. 4C and D).

In *K19-C2mE* mice at the age of 30 weeks, Alcian blue staining showed that the development of metaplastic changes was significantly less prominent in *Nrdc*^−/−^ mice compared with *Nrdc*^+*/*+^ mice (Fig. 4E and F). Together, these data indicate that the deletion of *Nrdc* leads to the suppression of metaplastic changes in the stomach.

### Formation of gastric tumors was suppressed by *Nrdc* deletion

**Figure 5:**
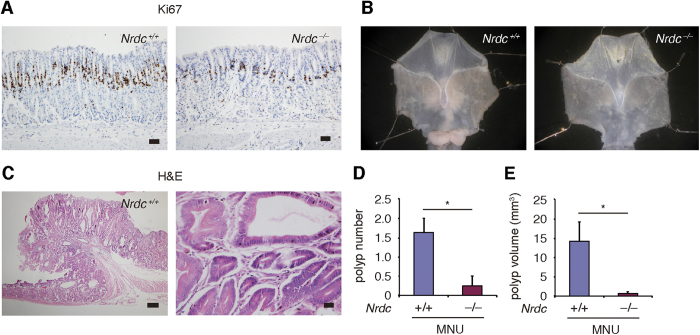
Formation of gastric tumors in *Nrdc*^+/+^ and Nrdc^−/−^ mouse stomachs. (**A**) Ki67 immunostaining in *Nrdc*^+*/*+^ and *Nrdc*^−/−^ mouse stomachs with the forced PGE_2_ expression. Bars = 50 μm. (**B**) Macroscopic views of the stomachs of *Nrdc*^+*/*+^ and *Nrdc*^−/−^ mice treated with N-methyl-N-nitrosourea (MNU). (**C**) H&E staining of gastric tumor in *Nrdc*^+*/*+^ mouse. Bars = 200 μm (left) and 20 μm (right). (**D**) Polyp number in the stomachs of *Nrdc*^+*/*+^ and *Nrdc*^−/−^ mice treated with MNU. *P < 0.05. (**E**) Polyp volume of *Nrdc*^+*/*+^ and *Nrdc*^−/−^ mouse gastric tumors established by MNU treatment. *P < 0.05.

Chronic gastritis and metaplastic changes are associated with the development of gastric cancers. In both *Helicobacter felis* infection and the *K19-C2mE* chronic gastritis mouse models, we noticed that the increase in the number of Ki67-positive cells in the gastric glands of *Nrdc*^+*/*+^ mice was not remarkable in *Nrdc*^−/−^ mice (Fig. 5A). Based on this finding, we hypothesized that the formation of gastric tumors may also be suppressed in *Nrdc*^−/−^ mice. Because it requires a long period to develop tumors in *Helicobacter felis*-infected mouse stomach, we administered a chemical carcinogen to rapidly induce mouse gastric tumors. Administration of N-methyl-N-nitrosourea (MNU) causes polypoid tumors in the gastric antrum with inflammatory reactions in the stroma^19^,^20^,^21^. In *Nrdc*^+*/*+^ mice, MNU treatment resulted in polyp formation in the gastric antrum (Fig. 5B and C). In *Nrdc*^−/−^ mice, the number and burden of gastric tumors was dramatically reduced compared with that in *Nrdc*^+*/*+^ mice (Fig. 5B,D and E). Thus, deletion of *Nrdc* attenuates gastric tumorigenesis induced by MNU.

## Discussion

In the present study, we demonstrated that Nrdc crucially regulates gastric inflammation caused by *Helicobacter felis* infection or forced expression of PGE_2_. Metaplastic changes following gastric inflammation were suppressed by the deletion of *Nrdc*. The deletion of *Nrdc* significantly suppressed chemically-induced tumorigenesis of the stomach.

*Helicobacter pylori*-induced gastritis is a primary inflammatory disorder of the human stomach, affecting about half of the global population^1^. Recent improvements in the hygienic environment have reduced the rate of *Helicobacter pylori* infection; however, other causative factors such as non-steroidal anti-inflammatory drugs or autoimmunity also contribute to the development of acute and chronic gastritis^22^. Regardless of the causative factors, chronic inflammation of the gastric mucosa can slowly progress to mucosal atrophy, induce metaplastic changes, and finally cause gastric cancer. In addition to the treatment of acute gastritis that may result in hemorrhagic complications, detection and therapeutic intervention in the early stages of the developmental cascade from chronic gastritis to gastric cancer is important. Therefore, it would be helpful to understand the molecular basis of chronic inflammation in the stomach.

Chronic inflammation is regulated by a feedback loop consisting of diverse inflammatory cytokines, and is closely associated with the recruitment of inflammatory cells in tissues. We previously demonstrated that the deletion of *Nrdc* critically suppresses ectodomain shedding, activation of TNF-α, and the production of other inflammatory cytokines in gastric cancer cells^14^. We also showed that the deletion of *Nrdc* significantly suppresses mouse steatohepatitis with attenuated inflammatory cytokine production and reduced infiltration of inflammatory cells^23^. Therefore, in this study, we examined the undetermined *in vivo* role of Nrdc during the developmental process of chronic gastritis, metaplastic changes, and gastric tumors.

To mimic chronic infection and inflammation in the human stomach caused by *Helicobacter pylori,* we used a *Helicobacter felis*-infected mouse model. Mucosae of the gastric corpus were thinner in *Nrdc*^−/−^ than in *Nrdc*^+*/*+^ mice, and the inflammation score was less severe in *Nrdc*^−/−^ mice. Infiltration of neutrophils and macrophages was decreased in *Nrdc*^−/−^ mice concomitantly with attenuated expression of inflammatory cytokines. Although infiltration of inflammatory cells such as neutrophils may not be critical for the development of *Helicobacter felis*-induced gastritis^24^, this pattern of cytokine expression is consistent with our previous data obtained from a human gastric cancer cell line^14^. Notably, levels of IL-1α, IL-1β, IL-6, and IL-12, which are the main contributors to human chronic gastritis, were decreased in *Nrdc*^−/−^ mice. Thus, the deletion of *Nrdc* attenuates gastric inflammation, indicating that Nrdc also plays a role in the intravital stomach. It is also important that the deletion of *Nrdc* attenuated inflammation caused by the forced expression of PGE_2_. This indicated that the deletion of *Nrdc* attenuates gastric inflammation regardless of the cause of inflammation, and a global therapeutic approach may be possible by targeting Nrdc.

We next questioned whether Nrdc plays a role in metaplastic changes and tumorigenesis. In *Helicobacter felis*-infected mice, metaplastic changes develop in the gastric corpus, and progress to mucous metaplasia that can be stained with Alcian blue^25^,^26^. In the present study, metaplastic changes induced by *Helicobacter felis* infection, or forced expression of PGE_2_, were significantly attenuated by the deletion of *Nrdc*. More importantly, gastric tumor formation was remarkably suppressed by the same genetic change. It is now becoming clear that the tumor microenvironment, which is orchestrated by inflammatory cells, is an indispensable participant during a wide-range of tumor developmental processes^27^.

Inflammation plays decisive roles at different stages of tumor development, including initiation, promotion, malignant conversion, invasion, and metastasis^28^. We previously reported that NRDC is highly expressed in the epithelium of human gastric cancer tissues^14^. Knockdown of *NRDC* attenuates gastric cancer cell growth both *in vitro* and in a xenograft model. These previous data showed that TNF-α secreted from gastric cancer cells themselves initiates a feedback loop to enhance inflammatory cytokine expression.

As for the pivotal role of Nrdc *in vivo*, we previously showed in a mouse steatohepatitis model that the production of inflammatory cytokines and recruitment of inflammatory cells are significantly suppressed concomitantly with the suppression of TNF-α release by the deletion of *Nrdc*^23^. Although we did not again examined the shedding status of TNF-α in mouse stomach in the present study, the remarkable suppressive effect of *Nrdc* deletion on inflammation may lead to the suppression of metaplastic changes and tumor formation. In this respect, it is worth noting that metaplastic changes were detected in *Nrdc*^−/−^ mice, albeit milder than in *Nrdc*^+*/*+^ mice, and that gastric tumor formation was almost completely blocked in *Nrdc*^−/−^ mice. This discrepancy indicates a possibility that formation of gastric tumors by MNU treatment requires inflammatory responses, and that metaplastic changes may be regulated at least partly by factors other than Nrdc. To determine the exact mechanisms underlying how Nrdc coordinates inflammation, metaplastic changes, and tumor formation, further studies are required. However, the findings obtained in available studies, including the current work, suggest that a global therapeutic approach against various gastric disorders may be possible by targeting NRDC.

## Methods

### Animal models

Generation of the *Nrdc* knockout mouse with a CBA background was described previously^15^. Animals were housed under specific pathogen-free conditions at the Animal Facilities of Kyoto University. All animal experiments were performed in accordance with institutional guidelines. The Review Board of Kyoto University granted ethical permission for this study, and The Kyoto University Animal Experimentation Committee approved the experimental protocol. For the infection by *Helicobacter felis*, suspending water containing *Helicobacter felis* was gavaged to *Nrdc*^+*/*+^ and *Nrdc*^−/−^ mice for three days according to the previous report^16^. *K19-C2mE* mice were generated as previously described^16^. To analyze chemically-induced gastric tumorigenesis, mice at the age of 6 weeks were administered MNU (Sigma-Aldrich, St. Louis, MO, USA) in drinking water at 240 ppm on alternate weeks for five weeks as described previously^19^.

### Histological and immunohistochemical analyses

Mouse stomach was resected, fixed in 4% buffered paraformaldehyde solution, embedded in paraffin, and cut into sections 5-μm thick. For immunostaining, the sections were incubated overnight with the primary antibodies at 4 °C, after which the secondary antibodies were added. The primary antibodies used were rat anti-F4/80 (Abcam, Cambridge, MA, USA), rat anti–Gr-1 (eBioscience, San Diego, CA, USA), sheep anti-pepsinogen II (Abcam), mouse anti-H^+^/K^+^-ATPase α subunit (MBL, Nagoya, Japan), mouse anti-Muc5AC (Abcam), mouse-anti-spasmolytic polypeptide (TFF2) (R&D Systems, Minneapolis, MN, USA), and rat anti-Ki67 (Dako, Glostrup, Denmark). All immunohistochemical analyses were performed with immunoglobulin isotype controls. For Alcian blue staining, deparaffinized sections were incubated with Alcian blue solution for 30 minutes, followed by counterstaining with Nuclear Fast Red. To determine the differentiation status of the gastric mucosa under physiological conditions, stained cells were counted in 10 randomly selected gastric glands per mouse in 6 *Nrdc*^+*/*+^ and 4 *Nrdc*^−/−^ mice. In *Helicbacter felis* infection experiments, we used 12 *Nrdc*^+*/*+^ and 6 *Nrdc*^−/−^ mice. In addition, 6 *Nrdc*^+*/*+^*; K19-C2mE* and 3 *Nrdc*^−/−^*; K19-C2mE* mouse samples were subjected to the analyses. Using these mice, to analyze histology and count inflammatory cells, eight high power field sections from each mouse were selected randomly. To investigate gastric tumorigenesis, 12 *Nrdc*^+*/*+^ and 6 *Nrdc*^−/−^ mice were treated with MNU. Inflammation scores were determined according to the previous report^24^.

### Real-time quantitative reverse transcription-polymerase chain reaction (qRT-PCR)

Total RNA was extracted using Trizol (Life Technologies, Carlsbad, CA, USA). Single-strand complementary DNA was synthesized using a First Strand SYBR Green Master Mix (Roche Applied Science, Basel, Switzerland). qRT-PCR was performed using FastStart SYBR Green Master (Roche Applied Science) and the LightCycler 480 system (Roche Applied Science). Values are expressed as arbitrary units relative to the expression of glyceraldehyde 3-phosphate dehydrogenase (GAPDH) expression. The primer sets used were: interleukin (IL)-1α-forward, CTGATGAAGCTCGTCAGGCAG; IL-1α-reverse, TGGTGCTGAGATAGTGTTTGTC; IL-1β-forward, GCAACTGTTCCTGAACTCAACT; IL-1β-reverse, ATCTTTTGGGGTCCGTCAACT; IL-6-forward, TAGTCCTTCCTACCCCAATTTCC; IL6-reverse, TTGGTCCTTAGCCACTCCTTC; IL-12-forward, ACTCTGCGCCAGAAACCTC; IL-12-reverse, CACCCTGTTGATGGTCACGAC; Cxcl1-forward, CTGGGATTCACCTCAAGAACATC; Cxcl1-reverse, CAGGGTCAAGGCAAGCCTC; Ccl2-forward, ATCCACGGCATACTATCAACATC; Ccl2-reverse, CAAGGCTCACCATCATCGTAG; Gapdh-forward, AGGTCGGTGTGAACGGATTTG; and Gapdh-reverse, TGTAGACCATGTAGTTGAGGTCA. In each experiment, 3–6 samples were subjected to the reactions.

### Statistical analyses

Results are expressed as means ± standard error unless stated otherwise. Differences between treatments, groups, and strains were analyzed by the two-tailed Student’s *t*-test.

## Additional Information

**How to cite this article**: Kimura, Y. *et al*. Nardilysin regulates inflammation, metaplasia, and tumors in murine stomach. *Sci. Rep.* **7**, 43052; doi: 10.1038/srep43052 (2017).

**Publisher's note:** Springer Nature remains neutral with regard to jurisdictional claims in published maps and institutional affiliations.
